# Regional Differences in Colorectal Cancer Mortality Between 2000 and 2013 in Republic of Korea

**DOI:** 10.2188/jea.JE20170331

**Published:** 2019-10-05

**Authors:** Hyeong Taek Woo, Jin Ah Sim, Jonghoon Mo, Young Ho Yun, Aesun Shin

**Affiliations:** 1 Department of Preventive Medicine, Seoul National University College of Medicine, Seoul, Republic of Korea; 2 Institute of Health Policy and Management, Medical Research Center, Seoul National University, Seoul, Republic of Korea; 3 Department of Psychology, Seoul National University College of Social Science, Seoul, Republic of Korea; 4 Department of Family Medicine, Seoul National University College of Medicine, Seoul, Republic of Korea; 5 Cancer Research Institute, Seoul National University, Seoul, Republic of Korea

**Keywords:** colorectal cancer (CRC), mortality, geographic difference, health geographical disparities

## Abstract

**Objective:**

Colorectal cancer (CRC) is the fourth most common site for cancer death in the Republic of Korea. The aim of this study was to describe the trends of colorectal cancer mortality by region.

**Methods:**

CRC mortality trends in Republic of Korea were described by region using a *Joinpoint* regression model in both sexes. The annual percent changes (APCs) were calculated for each segment. Visualization of the changes in mortality rate of colorectal cancer death rates by 16 geographic areas in both sexes between 2000–2004 and 2009–2013 were also conducted.

**Results:**

CRC mortality rates of men showed decreasing trend after increase in Daegu, Gyeongsangnam-do, and Chungcheongbuk-do between 2000 and 2013 based on the joinpoint model, while Gwangju, Jeollabuk-do, Jeollanam-do, and Gyeongsangbuk-do showed increase in CRC mortality during the same period. For women, CRC mortality of Seoul, Incheon, Daejeon, and Gyeongsangnam-do started to decrease in 2005, 2003, 2007, and 2006, respectively. The mortality rate for CRC in the eastern regions, which had relatively low rates of CRC among men in 2000 through 2004, reached a level similar to that in the northwestern regions of 2009 through 2013, while the highest CRC mortality rates in women was observed in Chungcheongbuk-do.

**Conclusions:**

Reduction in CRC mortality varied across 16 metropolitan cities and provinces in men, and the visualization pattern showed that the east side of South Korea had the least progress in mortality reduction.

## INTRODUCTION

Colorectal cancer (CRC) is the third leading cause of cancer death in both sexes in worldwide^1^^–^^3^ and the fourth most common cause of cancer death in the Republic of Korea, with crude rates of 18.5/100,000 for men and 13.8/100,000 for women in 2012.^4^ Mortality rates of CRC have been decreasing in countries in Europe, Oceania, and Asia for the past several decades.^5^^–^^7^ This decrease may be affected by increased uptake of CRC screening^6^^,^^8^^,^^9^ or differences in socioeconomic status.^10^^–^^12^

In previous studies, it has been projected that CRC mortality could be reduced with appropriate interventions,^13^^,^^14^ and deliberate public health action is being implemented in selected regions to accomplish the goal.^15^^–^^18^ Besides, disparities by geographical regions contribute to variation of CRC mortality.^9^^,^^11^^,^^19^ The geospatial approach describing the trends of CRC mortality would be highly useful to better understand the factors that contribute to differences in the overall CRC outcomes by region.^5^^,^^20^

In the Republic of Korea, decrease in CRC mortality was observed among women since 2004, whereas mortality of men was stabilized since 2002.^2^ However, the extent to which these decreases varies by sex and its influence on the regional characteristics of Korean CRC death rates are not documented in previous studies. Although the wide geographical variation in the mortality of CRC is evident, there was lack of evidence assessing regional differences with consideration of age and sex.

To this end, the present study aimed to explore the potential differences in trends of CRC mortality by geographical area and sex in the Republic of Korea. Therefore, we first analyzed CRC mortality trends in the Republic of Korea from 2000 through 2013 by province and sex; then, we examined the change in geographic visualization patterns of rates for two time intervals, 2000–2004 and 2009–2013.

## METHODS

### Materials

Age-specific mortality rate for CRC data from 2000 through 2013 for the 16 metropolitan cities and provinces (1 Special city, 6 metropolitan cities, 8 provinces, and Jeju Special Self-Governing Province) were obtained from the Korean Statistics Information Service (KOSIS) database as reported in the “Cause of Death” section of the statistical database. Mortality for cancers of the colon, rectum, and anus (ICD-10 code C18–C21) were extracted using the computer network (KOSIS, e-Nara Indicator) and standardized using the mid-year population of the Republic of Korea for 2005 and expressed per 100,000 populations.^21^^,^^22^

### CRC mortality and trends

CRC mortality trends in the Republic of Korea by region were described using a *Joinpoint* regression model to determine at which year the linear trend changed significantly in each of men and women. The *Joinpoint* regression models were fitted with a series of joined straight lines on a log scale to the annual age-standardized rates (ASRs). For each model, we allowed a maximum of 2 join-points for each area. The annual percent changes (APCs), which are the slopes of the change in death rates, were calculated for each segment. For each region, we also calculated the change in death rates between the 2000–2004 and 2009–2013 using rate ratio (2000–2004 to 2009–2013), with 95% confidential interval in both sexes. All statistical tests were two sided (*P* < 0.05). Joinpoint Regression Program version 4.2.0.1 (Statistical Methodology and Applications Branch, Surveillance Research Program, National Cancer Institute, Rockville, MD, USA) was used for statistical analysis (https://surveillance.cancer.gov/joinpoint/download).

### Geographic patterns of CRC death rates

To visualize the changes in mortality rate by geographic patterns of CRC death rates in both sexes between 2000–2004 and 2009–2013, we used regional maps using Korea ‘map shp’ from the Statistical Geographic Information Service with R version 3.1.0 (R Foundation for Statistical Computing, Vienna, Austria) (http://cran.r-project.org) Rgdal package (R for Geospatial Data Abstraction Library: https://r-forge.r-project.org/projects/rgdal/). The source map was driven by the Republic of Korea shp (Shape file) from SGIS. For each time interval, age-standardized rates for each state were sorted in descending order of rates and grouped into eight categories. The highest and lowest categories are approximately the 10th and 90th percentiles, whereas the remaining regions were divided into six equivalent groups. The color gradient in the maps reflect the CRC burden, with regions with the highest CRC mortality rate assigned the darkest color.

## RESULTS

**Figure 1.**
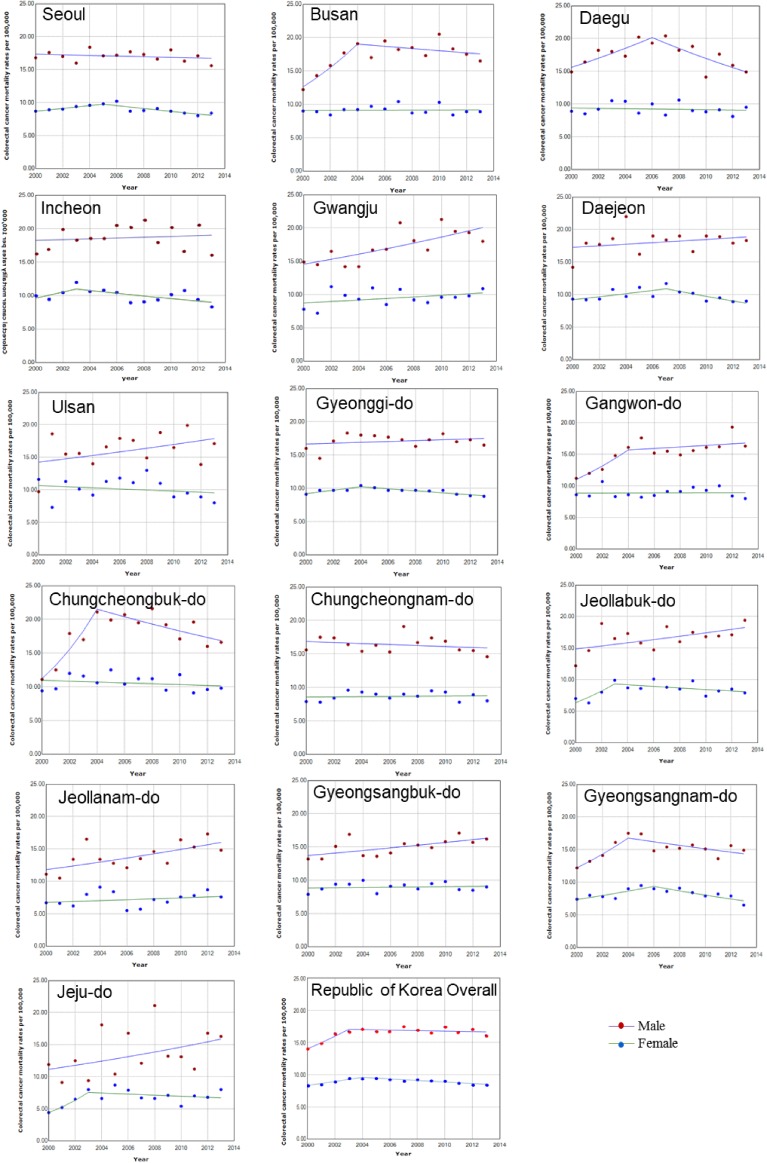
Trends in colorectal cancer mortality for each selected province, 2000–2013

**Figure 2.**
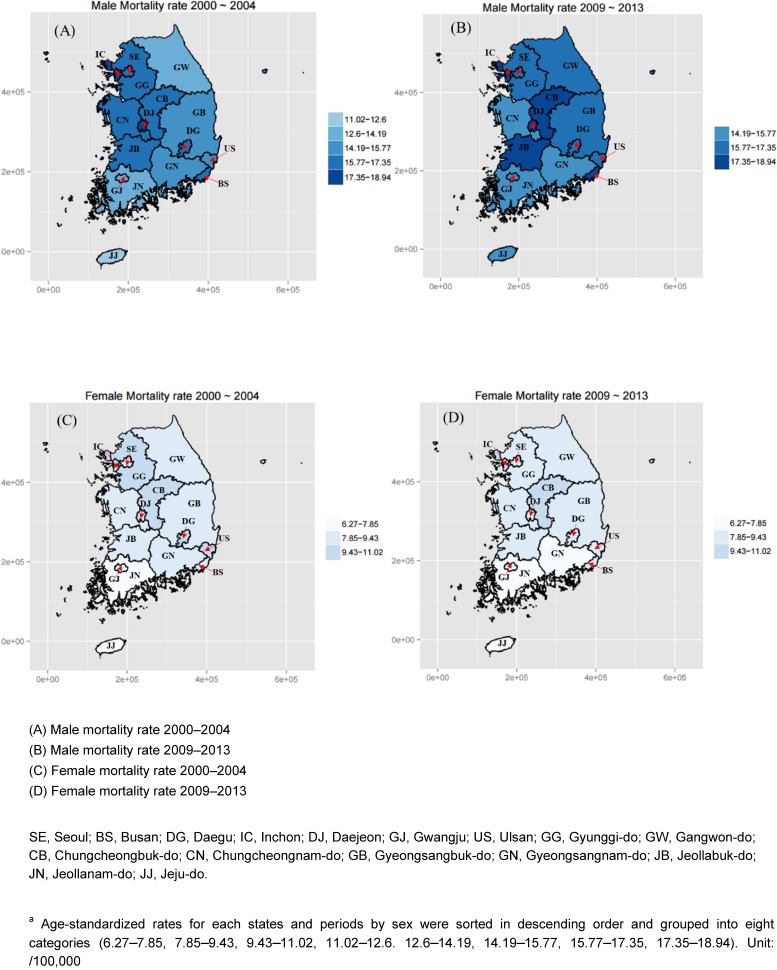
Colorectal cancer age-standardized mortality rates^a^ in 16 provinces, 2000–2004 to 2009–2013

Although the CRC mortality has been stabilized in men since 2003 nationwide, the mortality kept increasing until 2013 in Gwangju, Jeollabuk-do, Jeollanam-do, and Gyeongsangbuk-do, with significant APCs (Table 1 and Figure 1). Nationwide, the APCs in CRC death rates of women started to decrease from 2004 (Table 2 and Figure 1). According to joinpoint regression among women, mortality decreased significantly in Seoul since 2005, in Incheon since 2003, in Daejeon since 2007, and in Gyeongsangnam-do since 2006. Figure 2 illustrates the ASR of CRC mortality during 2000–2004 and 2009–2013 in both sexes by geographical region. During 2000–2004 in men, the highest CRC mortality rates were observed in metropolitan areas, such as Daejeon, Incheon, and Seoul, and the lowest mortality rates were observed in Gangwon-do, Jeollanam-do, and Jeju-do. During 2009–2013, the highest mortality rates in men were found in Gwangju and Incheon. Specifically for men, the west sides of South Korea showed higher mortality rate in 2000–2004; however, in 2009–2013, the mortality rate of CRC in the eastern regions increased, reaching a level similar to that in the western regions.

**Table 1.** Colorectal cancer (CRC) mortality per 100,000 and trends among men in 16 provinces, Republic of Korea, 2000–2013

Provinces (2010 Population size)	Age standardized mortality (2000–2013)														Mortality Rate (Male)		Rate ratios (95% CI)			Trend 1		Trend 2	
	00	01	02	03	04	05	06	07	08	09	10	11	12	13	2000–2004	2009–2013	RR	Lower CI	Upper CI	Years	APC	Years	APC
Republic of Korea	14	14.8	16.4	16.7	17.1	16.7	16.7	17.4	16.9	16.5	17.4	16.6	17	16.1	15.80	16.72	1.06	0.90	1.25	2000–2003	6.7^*^	2003–2013	−0.2
Seoul (4,726,028)	16.8	17.6	17.0	16.0	18.4	17.1	17.2	17.7	17.3	16.6	18.0	16.3	17.1	15.6	17.28	16.74	0.97	0.87	1.09	2000–2013	−0.28		
Busan (1,664,678)	12.2	14.3	15.8	17.7	19.1	17.0	19.5	18.2	18.5	17.3	20.5	18.3	17.5	16.5	16.05	17.94	1.12	0.93	1.34	2000–2004	10.81^*^	2004–2013	−0.9
Daegu (1,204,428)	14.9	16.4	18.2	18.0	17.3	20.2	19.3	20.4	18.2	18.8	14.1	17.6	15.9	14.9	17.03	16.35	0.96	0.76	1.21	2000–2006	4.36	2006–2013	−4.22^*^
Incheon (1,315,562)	16.2	16.9	19.9	18.2	18.6	18.5	20.6	20.3	21.3	18.0	20.2	16.7	20.5	16.1	18.11	18.45	1.02	0.82	1.27	2000–2013	0.32		
Gwangju (724,161)	14.9	14.5	16.5	14.2	14.2	16.7	16.8	20.8	18.1	16.7	21.3	19.5	19.3	18.0	14.98	18.94	1.26	0.93	1.73	2000–2013	2.49^*^		
Daejeon (745,150)	14.2	17.9	17.7	18.6	22.0	16.2	19.0	18.4	19.0	16.6	19.0	18.9	17.9	18.3	18.31	18.23	1.00	0.74	1.34	2000–2013	0.68		
Ulsan (550,869)	9.7	18.6	15.5	15.6	14.0	16.6	17.9	17.6	14.9	18.8	16.5	19.9	13.9	17.1	14.80	17.19	1.16	0.75	1.79	2000–2013	1.76		
Gyeonggi-do (5,599,570)	16.0	14.5	17.1	18.3	18.0	17.9	17.7	17.3	16.3	17.3	18.2	17.0	17.3	16.5	17.00	17.27	1.02	0.91	1.14	2000–2013	0.39		
Gangwon-do (735,075)	11.2	12.0	12.6	14.8	16.1	17.6	15.2	15.5	14.9	15.6	16.1	16.2	19.3	16.3	13.48	16.85	1.25	0.98	1.60	2000–2004	9.25^*^	2004–2013	0.75
Chungcheongbuk-do (748,622)	11.1	12.5	17.9	17.0	21.1	19.9	20.7	19.5	21.6	19.2	17.1	19.6	16.0	16.6	16.04	17.63	1.10	0.86	1.41	2000–2004	17.28^*^	2004–2013	−2.68^*^
Chungcheongnam-do (1,007,454)	15.6	17.5	17.4	16.4	15.4	16.3	15.3	19.1	16.7	17.4	16.9	15.6	15.5	14.6	16.48	15.73	0.95	0.78	1.17	2000–2013	−0.45		
Jeollabuk-do (867,630)	12.2	14.6	18.9	16.5	17.3	15.8	14.7	18.4	16.0	17.5	16.8	16.9	17.1	19.4	16.00	17.59	1.10	0.89	1.36	2000–2013	1.60^*^		
Jeollanam-do (845,952)	11.1	10.5	13.4	16.5	13.4	12.8	12.1	13.5	14.6	12.8	16.4	15.3	17.3	14.8	12.94	15.36	1.19	0.96	1.47	2000–2013	2.37^*^		
Gyeongsangbuk-do (1,281,510)	13.2	13.2	15.1	16.9	13.7	13.6	14.1	15.5	15.3	14.9	15.8	17.1	15.7	16.2	14.50	16.00	1.10	0.92	1.32	2000–2013	1.35^*^		
Gyeongsangnam-do (1,562,686)	12.2	13.2	14.1	16.1	17.5	17.4	14.8	15.4	15.2	15.7	15.1	13.6	15.6	14.9	14.84	15.01	1.01	0.83	1.23	2000–2004	8.25^*^	2004–2013	−1.71^*^
Jeju-do (261,521)	11.9	9.1	12.5	9.4	18.1	10.4	16.8	12.1	21.1	13.2	13.1	11.2	16.8	16.3	12.54	14.42	1.15	0.70	1.90	2000–2013	2.76		

Mortality rate is per 100,000, age adjusted to the 2005 Republic of Korea standard population.

APC, Annual Percent Change; CI, Confidential Interval with IACR method; RR, Rate Ratios.

^*^APC is significantly different from zero at alpha = 0.05.

**Table 2.** Colorectal cancer (CRC) mortality per 100,000 and trends among women in 16 provinces, Republic of Korea, 2000–2013

Provinces (2010 Population size)	Age standardized mortality rate (2000–2013)														Mortality Rate (Female)		Rate ratios (95% CI)			Trend 1		Trend 2	
	00	01	02	03	04	05	06	07	08	09	10	11	12	13	2000–2004	2009–2013	RR	Lower CI	Upper CI	Years	APC	Years	APC
Republic of Korea	8.4	8.5	9	9.4	9.5	9.5	9.3	9.1	9.2	9.1	9.1	8.7	8.5	8.5	8.96	8.78	0.98	0.86	1.12	2000–2004	3.6^*^	2004–2013	−1.3^*^
Seoul (4,905,454)	8.7	8.9	9.0	9.4	9.6	9.8	10.2	8.7	8.8	9.1	8.7	8.4	8.0	8.4	9.17	8.58	0.94	0.76	1.15	2000–2005	2.4	2005–2013	−2.38^*^
Busan (1,728,513)	9.0	8.9	8.4	9.2	9.2	9.7	9.3	10.4	8.7	8.8	10.3	8.4	8.9	8.9	8.93	9.12	1.02	0.73	1.42	2000–2013	0.1		
Daegu (1,227,346)	8.9	8.5	9.2	10.5	10.4	8.6	10.0	8.3	10.6	9.0	8.8	9.1	8.1	9.5	9.59	8.99	0.94	0.64	1.38	2000–2013	−0.3		
Incheon (1,316,473)	10.0	9.5	10.5	12.0	10.6	10.8	10.5	9.0	9.1	9.4	10.2	10.8	9.4	8.3	10.56	9.64	0.91	0.62	1.34	2000–2003	4.58	2003–2013	−2.01^*^
Gwangju (741,982)	7.8	7.2	11.2	9.9	9.3	11.0	8.5	10.8	9.2	8.8	9.6	9.6	9.8	10.9	9.10	9.79	1.08	0.63	1.85	2000–2013	1.2		
Daejeon (745,008)	9.3	9.2	9.3	10.8	9.7	11.1	9.7	11.7	10.4	10.2	9.0	9.5	8.9	9.0	9.68	9.37	0.97	0.57	1.64	2000–2007	2.5	2007–2013	−3.74^*^
Ulsan (520,804)	11.6	7.3	11.3	10.1	9.2	11.3	11.8	11.1	13.0	11.0	8.9	9.5	8.9	8.0	9.91	9.31	0.94	0.46	1.92	2000–2013	−0.8		
Gyeonggi-do (5,596,483)	9.1	9.7	9.7	9.7	10.4	10.1	9.7	9.7	9.7	9.6	9.7	9.1	8.9	8.8	9.79	9.24	0.94	0.78	1.14	2000–2004	2.70^*^	2004–2013	−1.6
Gangwon-do (728,575)	8.6	8.4	10.7	8.3	8.6	8.2	8.5	9.1	9.1	9.8	9.3	10.0	8.4	8.0	8.90	9.10	1.02	0.67	1.55	2000–2013	0.1		
Chungcheongbuk-do (747,362)	9.4	9.7	12.0	11.6	10.6	12.5	10.4	11.2	11.2	9.5	11.8	9.1	9.6	9.8	10.73	9.99	0.93	0.63	1.39	2000–2013	−0.6		
Chungcheongnam-do (993,019)	7.9	7.8	8.4	9.6	9.3	9.0	8.4	9.0	8.7	9.5	9.3	7.8	8.9	8.0	8.63	8.58	0.99	0.69	1.43	2000–2013	0.2		
Jeollabuk-do (898,414)	7.0	6.3	8.0	9.9	8.7	8.6	10.1	8.8	8.5	9.8	7.4	8.2	8.5	7.9	8.03	8.38	1.04	0.70	1.56	2000–2003	13.7	2003–2013	−1.4
Jeollanam-do (882,797)	6.7	6.6	6.2	8.0	9.1	8.4	5.5	5.7	7.2	6.8	7.6	7.8	8.7	7.6	7.36	7.70	1.05	0.71	1.54	2000–2013	1.0		
Gyeongsangbuk-do (1,293,860)	7.9	8.7	9.4	9.4	10.0	8.0	9.1	9.3	8.7	9.5	9.8	8.6	8.5	9.0	9.10	9.11	1.00	0.74	1.35	2000–2013	0.2		
Gyeongsangnam-do (1,556,885)	7.4	8.0	7.8	7.5	9.0	9.5	9.0	8.6	9.1	8.4	7.9	8.2	7.9	6.5	7.98	7.81	0.98	0.70	1.38	2000–2006	4.10^*^	2006–2013	−3.77^*^
Jeju-do (266,890)	4.4	5.2	6.5	8.0	6.6	8.7	7.9	6.7	6.6	7.1	5.4	7.0	6.8	8.0	6.27	6.97	1.11	0.44	2.82	2000–2003	19.7	2003–2013	−1.2

Mortality rate is per 100,000, age adjusted to the 2005 Republic of Korea standard population.

APC, Annual Percent Change; CI, Confidential Interval with IACR method; RR, Rate Ratios.

^*^APC is significantly different from zero at alpha = 0.05.

In contrast, death rates of all cities and provinces decreased or were stable in women. The mortality rate ratios between 2000–2004 and 2009–2013 were the lowest in Incheon (0.91), followed by Chungcheongbuk-do (0.93), whereas Jeju-do (1.11) and Gwangju (1.08) showed the highest rate ratios, although none of the rate ratios were statistically significant.

## DISCUSSION

In this study, the data provided are the most recent regional information of CRC in South Korea from KOSIS. Mortality data of CRC in South Korea from 16 metropolitan cities and provinces are presented. Similar to other countries’ geographical studies,^5^^,^^23^ we analyzed temporal trends in age-standardized CRC death rates from 2000 through 2013 by geographical areas in both sexes and the change in geographic patterns of rates for two time intervals, 2000–2004 and 2009–2013. The study showed that significant regional differences in CRC mortality were shown in men, even in the rural areas (Gangwon-do, Jeollabuk-do, Jeollanam-do, and Gyeongsangbuk-do) where mortality increased from 2000 to 2013. In the Republic of Korea, cancer-related survival burden is expected to show a rapid growth with the increasing cancer incidence and mortality.^24^^–^^26^

We also found that the time trends of some provinces indicated that there were joinpoint around 2004. In the Republic of Korea, the age-standardized mortality rate of men for CRC increased from 1984 through 2003, then remained stable thereafter, while the CRC mortality rate in women started to decrease since 2004.^27^ Those findings have been affected by an underlying birth-cohort pattern: while the period effects also steadily increased until 2004 among both sexes,^2^ the cohort effect curve showed decreasing patterns since 1919, which resulted in the decreasing relative risk of CRC mortality in recent younger cohorts.

In addition, the national cancer screening program (NCPS) in Korea for CRC using fecal occult blood test (FOBT) began in 2004.^28^ People who are within the lower 50% of insurance premium bucket of the National Health Insurance Corporation (NHIC) are the targeted population for the NCSP. Therefore, introduction of a screening program may contribute to improved CRC survival and consequent reduction in mortality in Korea.^29^ In Korea, CRC incidence rapidly increased until 2011, then started to decrease in both men and women. Introduction of a national cancer screening program may have affected the reduction of mortality rate and transient increase in incidence due to finding of prevalent cases during the initial period of the screening program.

To the best of our knowledge, this is the first study to report on the regional differences in CRC mortality in Republic of Korea. In prior studies conducted in the United States, socio-economic status (SES) inequalities in CRC death by state^11^^,^^20^ were investigated using SEER cancer statistic data.^5^ Earlier studies suggested that there were significant geographic disparities in the United States for overall CRC mortality trends, and those differences are explained by the inequality factor of education^11^^,^^20^ or uptake of screening rates.^5^ In addition, in China, there was a study investigating CRC mortality calculated by area, sex, and age category groups.^23^

Considering our results with those of other studies, possible explanations for geographical disparities in CRC death rates in the Republic of Korea includes multiple factors, such as uptake of screening, as well as several known behavioral^30^^,^^31^ and lifestyle factors, such as physical inactivity and unhealthy dietary habits.^10^^–^^12^^,^^32^ From the previous studies, regions whose uptake of CRC screening was lower were more likely to have CRC cases with advanced stages^4^^,^^33^ and were related with less decrease in mortality rates compared with other regions.^5^ In this study, we did not analyze primary causative factors affecting CRC death rates according to the geographical patterns, leaving the possibility that this disparity can be explained by multiple factors due to geographical differences.^5^^,^^10^ While, among other lifestyle factors, obesity may increase the risk of CRC.^34^^,^^35^ Based on the data of NHIS, the top ranking of obesity prevalence in 2015 was observed in Jeju-do, Gangwon-do, Incheon, and Jeollado areas, which showed increasing rates of CRC from 2000–2004 to 2009–2013 in men.

Even if data on regional variation in CRC treatment are limited, CRC survivors residing in rural areas or poorer provinces are less likely to receive appropriate and timely treatment than those residing in urban or more affluent areas.^10^^,^^36^ From previous studies, people with lower SES may delay their treatment for CRC due to diverse factors, including lack of treatment-related knowledge, care burden, and limited medical accessibility.^10^^,^^37^ CRC patients residing in rural areas or poorer neighborhoods are less likely to receive adjuvant chemotherapy than patients residing in urban or more affluent areas.

A limitation of our study is the validity of death certificates, which can affect our results. The Cause of Death Statistics of Korea is primarily based on death certificates. In a previous study, overall accuracy of the Cause of Death Statistics was 91.9%.^38^ In addition, if there were no available death certificates, deaths were classified as unknown causes. Other previous studies identified that, overall, 18.3% of deaths were classified as unknown causes and proportion of unknown causes varied region to region.^39^ In other aspects, overall, as proportions of death certificates issued by physicians have steadily increased,^40^ the proportion of unknown cause of death has steadily decreased. This could contribute to little change in CRC mortality rate in most areas. However, since CRC is major neoplasm, its diagnosis is more likely to be fairly accurate.

The time of trends examined from 2000 through 2013 may also be limitation. Cause-specific mortality data was available from 1983, so we could calculated the yearly ASR per 100,000 based on the KOSIS from 1983 to 2013. However, because we wanted to look at the recent trends in the past 10 years, we only selected the data from 2000 to 2013. Therefore, in our study we chose the year of 2000 as the observation starting point. In addition, although we tried to explain the regional differences of CRC mortality with prevalence of known risk factors, such as smoking and alcohol consumption, and especially for screening, a further study to investigate the reasons for regional differences in trends of CRC mortality based on more descriptive evidence should be pursued.

Nevertheless, the main strength of this study is that we examined representative CRC mortality trends in Korea using nationwide data. Joinpoint regression usually needs a large sample size, although statistical power to detect a significant APC depends on a value size of APC and rate per population. In addition, this study is the first to show the geographical pattern of CRC death rates using visualization data. We found that the mortality rate for CRC in the eastern regions, such as Deagu after 2006, Chongchengbuk-do after 2004, and Gyeongsangnam-do after 2004, which had relatively lower rates than that of the men in 2000 to 2004, reached a level similar to that in the northwestern regions of 2009 to 2013. Northwestern regions, such as Incheon and Daejeon, showed stable and higher CRC over the observation period in men; Gwangju, Jeollabuk-do, Jeollanum-do, and Gyeongsamgbuk-do, which are located in the southwest, showed an increasing trend of CRC mortality. Generally, the regional disparity of CRC death rates decreased in men from 2000–2004 to 2009–2013.

In conclusion, regional variation in CRC mortality of men became less apparent between 2000–2013. Still, based on the APC analysis, CRC mortality has started to decline in Daegu since 2006 and in Chungcheongnam-do and Gyeongsangnam-do since 2004 in men. In women, the mortality rate of CRC started to decrease in 2005, 2003, 2007, and 2006 in Seoul, Incheon, Daejeon, and Gyeongsangnam-do, respectively.
